# Sequence of Chemotherapy May Not Impact Survival After Resection of Pancreatic Tail Adenocarcinoma

**DOI:** 10.1002/jso.28086

**Published:** 2025-01-13

**Authors:** Chase J. Wehrle, Jenny Chang, Abby Gross, Breanna Perlmutter, Robert Naples, Katherine Stackhouse, Toms Augustin, Daniel Joyce, Robert Simon, Andrea Schlegel, R. Matthew Walsh, Samer A. Naffouje, Alessandro Parente

**Affiliations:** ^1^ Department of General Surgery Cleveland Clinic Foundation Cleveland Ohio USA; ^2^ Institute of Liver Studies, King's College Hospital NHS Foundation Trust London UK

## Abstract

**Introduction:**

Pancreatic ductal adenocarcinoma (PDAC) of the body/tail is notably different than PDAC in the head of the pancreas. Surgery plus chemotherapy is known to improve outcomes for all PDAC. The sequence of this therapy is well studied in head cancers yet has never been evaluated systematically in relation to distal pancreatectomy (DP).

**Methods:**

Patients receiving DP for PDAC and who received chemotherapy were included. Patients were compared receiving neoadjuvant systemic therapy (NAST) only, adjuvant (AST) only, both NAST + AST, and who received total neoadjuvant therapy (TNT), defined as > 24 weeks NAST before DP. PSM was performed 1:1 between AST and each other group creating quadruplets of patients for analysis. Matching factors were determined by multivariate cox‐regression analysis of factors independently affecting survival. Survival was considered from diagnosis and from surgery to account for potential biases.

**Results:**

In total, 4677 patients were selected with 400 (8.6%) receiving TNT, 536 (11.5%) NAST, 3235 (69.2%) AST, and 506 (10.8%) NAST + AST. A total of 341 quadruplets were selected after PSM. There were no differences in comorbidities, T/N‐stage, retrieved or positive lymph nodes, and margin status after matching. Kaplan–Meier analysis showed no difference in median OS between the matched treatment groups (33.71 ± 2.07 vs. 35.22 ± 1.62 vs. 32.53 ± 3.31 vs. 37.88 ± 1.90, respectively; log‐rank *p* = 0.464). Five‐year OS was not different between the groups (21% vs. 18% vs. 20% vs. 25%, respectively; *p* = 0.501).

**Conclusion:**

The sequence of chemotherapy and surgery did not impact survival in distal PDAC. Providers should tailor an individualized approach designed to maximize the chance of completing both treatments.

**Abbreviations**
AST: adjuvant chemotherapy
DP: distal pancreatectomy
NAST: neoadjuvant systemic therapy
NCDB: National Cancer Database
OS: overall survival
PD: pancreaticoduodenectomy
PDAC: pancreatic ductal adenocarcinoma
TNT: total neoadjuvant therapy

## 1 Introduction

Pancreatic cancer is a highly lethal malignancy with outcomes made worse by the low rate of early detection [1]. The incidence of pancreatic ductal adenocarcinoma (PDAC), the most common histologic type, is increasing annually, making its treatment highly relevant to current oncologic practices [1, 2]. The optimal treatment for PDAC is surgical resection plus chemotherapy either before, after or both before and after pancreatectomy [3]. Surgical resection of PDAC follows a fairly stringent set of guidelines given the high morbidity of pancreatic resection coupled with the simultaneously high rate of disease recurrence after curative‐intent resection [4, 5]. Resectability is typically discussed separately for pancreatic head lesions, which require pancreaticoduodenectomy (PD, or a Whipple operation), vs. distal lesions, which can be treated with a distal pancreatectomy (DP) [6].

It is widely known that both chemotherapy and surgery combined improve outcomes for PDAC, yet the timing of chemotherapy relative to surgery remains highly debated [4, 5]. Some studies advocate the benefit to neoadjuvant chemotherapy for this purpose followed by resection, though others including randomized trials support equivalency of up‐front surgery [3, 7]. Meta‐analyses of randomized controlled trials generally advocate that borderline or un‐resectable PDAC benefits from neoadjuvant systemic therapy (NAST), yet there is no consensus for resectable disease [8]. However, studies tend to evaluate either only PDAC located in the pancreatic head, or collectively analyze head, body, and tail lesions. Meta‐analyses of > 90 studies and > 250 000 patients have shown that pancreatic body/tail cancers have worse long‐term prognosis, highlighting how these two are likely to benefit from different management approaches [9].

No study has yet assessed how the timing of chemotherapy affects outcomes after resection of only pancreatic body and tail lesions. We aim to provide the first large‐scale assessment of the impact of timing of peri‐operative chemotherapy on outcomes of resection of PDAC located in the pancreatic body and tail.

## 2 Methods

### 2.1 Data Source and Patient Selection

The National Cancer Database (NCDB) for pancreatic malignancies in adult patients between 2004 and 2021 was used for this analysis. We applied a series of inclusion/exclusion criteria as follows:

- Only patients with PDAC histology.
- Only patients with PDAC in the body and tail of the pancreas.
- Only patients who had non‐metastatic disease who received a DP.
- Only patients who received systemic chemotherapy in addition to surgery were selected. This selection included patients who received systemic therapy in the neoadjuvant (i.e., NAST) or adjuvant setting (i.e., AST). NAST patients who received systemic therapy for ≥ 24 weeks were considered to have had total neoadjuvant therapy (i.e., TNT).
- Only patients with a complete report on nodal yield, margin status, and nodal staging were included.

After applying the selection criteria, we divided the population into four groups based on the sequence of systemic therapy to the surgical resection: (1) TNT, (2) NAST, (3) AST, and (4) NAST + AST. Therefore, receipt of perioperative chemotherapy was set as the primary exposure variable. The following patient‐level characteristics were evaluated: age, race (White, Black, and other), Charlson/Deyo comorbidity score, year of diagnosis, insurance status (Medicaid/Medicare, Private Insurance, and Uninsured), zip code, level of education status, nodal status (N0, N1, and N2), tumor grade/differentiation (well, moderate, poor, and anaplastic), and lymphovascular invasion (absent and present).

### 2.2 Study Outcomes

In the four cohorts which were analyzed separately, the overall survival (OS) was studied for between the groups as the primary outcome. Survival was considered from two points. First, we considered survival from the earliest date in the database representing the time of diagnosis. However, the NCDB only includes surgical cases, and thus patients who received NAST or TNT have a mandatory survival advantage of 6–12 weeks vs. those who receive their chemotherapy after surgery. They may also have an additional benefit of successfully completing courses of chemotherapy and remaining surgically resectable, highlighting positive tumor biology. To address these concerns, we do provide an analysis that re‐defines survival from the time of surgery and again compared groups by chemotherapy. However, it is our intention to assess the optimal treatment sequence for a patient from their first presentation, which requires analysis from the presentation to completely assess.

### 2.3 Statistical Analysis

Baseline demographic and clinical characteristics were compared between the groups using conditional logistic regression for categorical variables and mixed effect modeling for continuous variables. A Cox regression survival analysis was performed to identify independent predictors of OS excluding the systemic therapy sequence. A conditional backward stepwise approach with *p* threshold of < 0.05 for inclusion and > 0.1 for exclusion in each step was employed. Analysis was adjusted for hospital and patient‐level information including age, sex, CDCC score, insurance status, and education level. We considered AST to be the standard group given its largest size and constructed multivariable binary logistic regressions using the significant OS predictors for the likelihood of receiving a certain sequence of chemotherapy over the standard AST. We repeated this regression three times using AST vs. TNT, AST vs. NAST, and AST vs. NAST + AST and calculated the propensity score for each model. 1:1 matching was performed between AST and each group using the nearest neighbor method and a 0.05 caliper width. Patients with a mutual reference match from the AST groups were identified to identify the final matched quadruplets in a 1:1:1:1 ratio.

The clinical and demographic characteristics were compared between the groups to ensure adequate calibration and balance with a goal of standard difference (SD) < 0.05 and *p* value < 0.05. The matched groups were then used to create a Kaplan–Meier OS analysis. Median OS was compared using the log‐rank test. Five‐year OS were compared using Fischer's exact test. SPSS v29 was the software used in this study.

## 3 Results

### 3.1 Baseline Characteristics of the Cohorts

After applying the inclusion/exclusion criteria, 4677 patients were selected. The mean age was 65.9 ± 15.8 years and 2253 (48.2%) were males. In total, 2567 (54.9%) had T3 tumors and 2225 (47.6%) had node‐negative disease. Surgical margins were positive in 867 (18.5%) of cases. Regarding the sequence of systemic therapy, 400 (8.6%) patients received TNT, 536 (11.5%) received NAST, 3235 (69.2%) received AST, and the remaining 506 (10.8%) received NAST + AST. Neoadjuvant radiation was offered to 499 (10.9%) patients, whereas 1246 (26.6%) received adjuvant radiation and 2932 (62.7%) did not receive any radiation. Median follow‐up was 26.3 months. Table 1 summarizes the demographic and clinical characteristics of the selected patient population.

**Table 1:** Summary of the demographic and clinical characteristics of the selected patients who underwent a distal pancreatectomy for non‐metastatic pancreatic ductal adenocarcinoma and perioperative systemic therapy.

*N*		4677
Age (years)	Mean ± SD	65.9 ± 15.8
	Median	67
Sex	Male	2253 (48.2%)
	Female	2424 (51.8%)
Race	White	3935 (84.1%)
	Black	517 (11.1%)
	Other	225 (4.8%)
Charlson score	0	2977 (63.7%)
	1	1202 (25.7%)
	2	325 (6.9%)
	3+	173 (3.7%)
Pathologic T stage	T1	708 (15.1%)
	T2	1141 (24.4%)
	T3	2567 (54.9%)
	T4	129 (2.8%)
	Tx	132 (2.8%)
Pathologic N stage	N0	2225 (47.6%)
	N1	1768 (37.8%)
	N2	684 (14.6%)
Examined nodes	Mean ± SD	15.8 ± 9.8
	Median	14
Positive nodes	Mean ± SD	1.6 ± 2.4
	Median	1
Margins	Negative	3810 (81.5%)
	Positive	867 (18.5%)
Systemic therapy	Total neoadjuvant	400 (8.6%)
	Neoadjuvant	536 (11.5%)
	Adjuvant	3235 (69.2%)
	Both	506 (10.8%)
Radiation	None	2932 (62.7%)
	Neoadjuvant	499 (10.7%)
	Adjuvant	1246 (26.6%)
Follow‐up	Mean ± SD	36.5 ± 31.1
	Median	26.3

A Cox regression analysis was performed utilizing all available variables except for the sequence of systemic therapy in the model. The final block demonstrated that age, Charlson score, T stage, N stage, number of retrieved nodes, and margin status were significant predictors of OS (Table 2). Comparison of baseline characteristics between the treatment groups showed significant differences in age, T stage, N stage, number of retrieved nodes, and margin status between the groups. Table S1 shows a summary of this baseline comparison between the groups before the match.

**Table 2:** Cox regression analysis for predictors of overall survival in the selected patient population.

	*β*	Hazard ratio [95% confidence interval]	*p*
Age	0.010	1.010 [1.006–1.013]	< 0.001*
Sex			
Male		Reference	
Female	−0.022	0.979 [0.913–1.049]	0.541
Race			
White		Reference	
Black	−0.087	0.917 [0.818–1.027]	0.134
Other	−0.224	0.800 [0.473–1.050]	0.122
Charlson score			
0		Reference	
1	0.118	1.125 [1.039–1.219]	0.004*
2	0.170	1.185 [1.034–1.358]	0.014*
3+	0.208	1.232 [1.026–1.478]	0.025*
Pathologic T stage			
T1		Reference	
T2	0.390	1.477 [1.290–1.692]	< 0.001*
T3	0.560	1.751 [1.547–1.983]	< 0.001*
T4	0.682	1.978 [1.577–2.481]	< 0.001*
Tx	0.645	1.906 [1.529–2.375]	< 0.001*
Pathologic N stage			
N0		Reference	
N1	0.347	1.414 [1.307–1.530]	< 0.001*
N2	0.607	1.835 [1.651–2.039]	< 0.001*
*N* of examined nodes	−0.013	0.987 [0.983–0.991]	< 0.001*
Margins			
Negative		Reference	
Positive	0.435	1.544 [1.418–1.682]	< 0.001*

*Note:* Bold values indicate statistically significance *p* < 0.05.

Therefore, 341 quadruplets were selected. We repeated the comparison between the selected groups which showed resolution of all the significant differences with SD and *p* values < 0.05. Table S2 shows the comparison between the treatment groups after the match.

### 3.2 Survival Analysis

**Figure 1:**
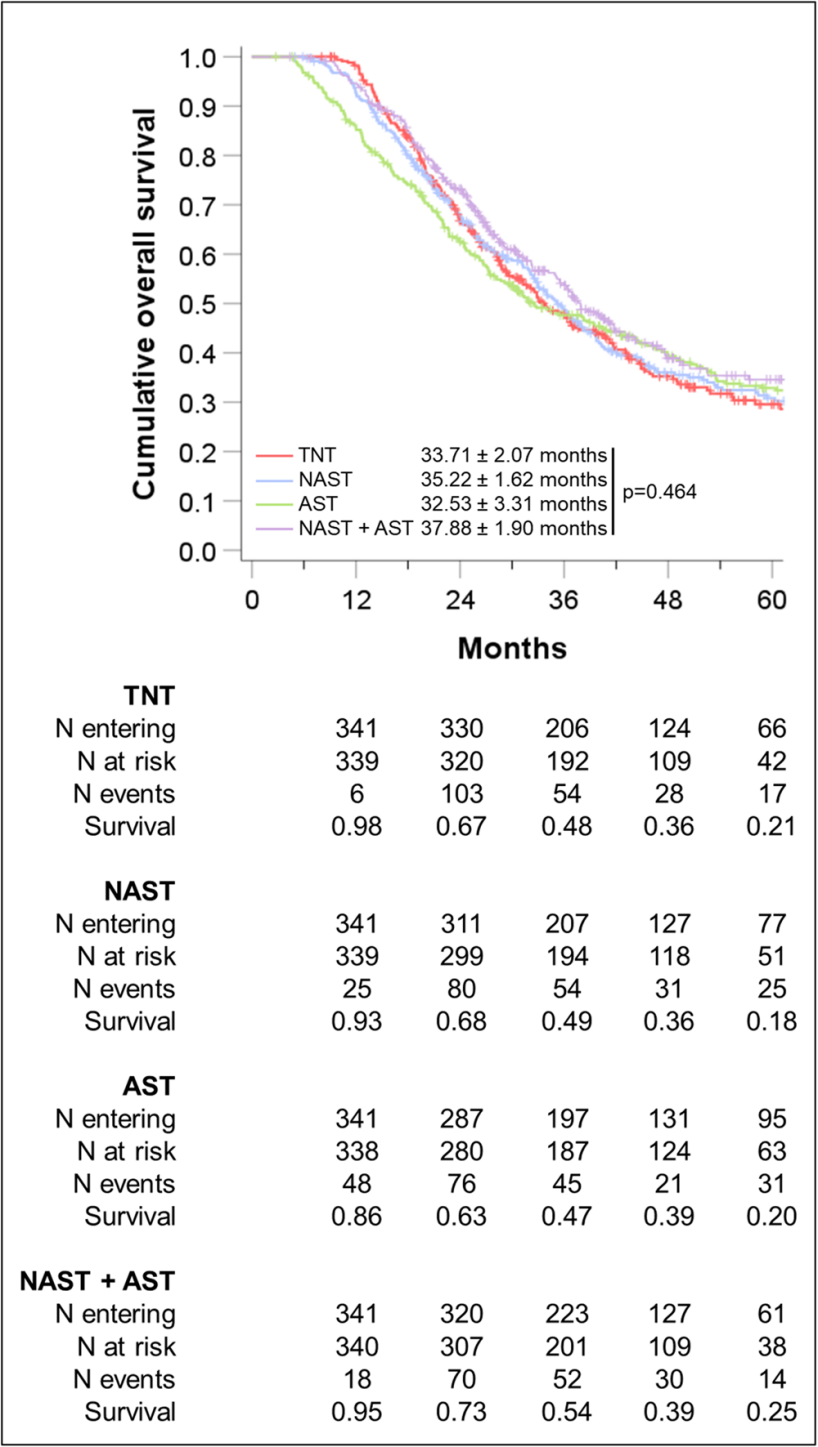
Kaplan–Meier plots for overall survival in the matched groups with survival defined from the first recorded date.

Kaplan–Meier analysis showed a significant reduction in median survival between groups, with the shortest median survival from the time of surgery found in the TNT group (25.8 ± 2.4 months) followed by NAST (27.7 ± 1.9 months), NAST + AST (31.93 ± 2.5 months) and the longest survival from surgery in the AST group (32.53 ± 3.3 months, *p* = 0.012). This does translate to an increase in actuarial 5‐year OS from 26% in the TNT group to 33% in the AST and NAST + AST groups [Figure 1].

Sensitivity analysis was performed defining survival from the point of surgery. This identified an increased median survival in patients receiving NAST + AST and AST‐alone vs. those receiving TNT or NAST [Supplement S3].

## 4 Discussion

This is the first study to specifically evaluate the sequence of chemotherapy relative to distal pancreatic resection for up‐front resectable pancreatic body and tail ductal adenocarcinoma. Most critically, by analyzing 4677 patients we demonstrate no difference in survival based on chemotherapy vs. surgical timing when considering survival from the point of diagnosis, which represents our ultimate goal to improve survival from the first patient presentation. The overall outcomes in all groups were expectedly poor, highlighting that radical shifts in the field might be necessary to achieve a significant change in patient outcomes. These findings might guide surgeons and oncologists alike in selecting an approach for each patient based on maximizing their likelihood of completing both chemotherapy and surgery as receipt of both factors is associated with optimal outcomes.

The receipt of both chemotherapy and surgery in the management of PDAC has been well established as improving outcomes over either approach individually [10, 11, 12, 13, 14, 15]. Completion of perioperative chemotherapy, however, remains a challenge for many patients secondary to drug toxicity, surgical complications, and socioeconomic and demographic factors [11]. Research has continued to debate the proper sequence of chemotherapy and surgery, including TNT, where all systemic therapy is given prior to resection, NAST then surgery, up‐front surgery then AST, or NAST followed by surgery and AST. In pancreatic head cancers, some form of NAST has been shown to be beneficial in borderline or un‐resectable disease, with AST offering additional benefits if patients qualify after surgical recovery [3, 4, 5, 7]. However, there is no clear consensus for distal cancers, which are less common, and more aggressive [9]. Studies such as the NORPACT trial support no benefit to neoadjuvant therapy, though this was not limited to distal cancers [16]. In the present study, we found that there was no difference in survival by the sequence of chemotherapy and surgery. This generally supports providers in selecting an approach most likely to achieve successful completion of both chemotherapy and surgical resection. In particular, a provider may consider a patient's relative likelihood of completing chemotherapy and decide whether earlier or later resection would be most likely to successfully complete both resection and chemotherapy, the combination of which are undoubtedly critical to achieving maximal survival. Unfortunately, granular data on rationale are not captured by the NCDB, and treatment selection rationale may have led to selection bias, therefore it is not possible to determine how the extent of competing biases might have altered the outcomes in this report.

We note that up to one‐third of patients do not receive chemotherapy after resection of PDAC, emphasizing importance of the timing of chemotherapy [17]. While DP ± splenectomy does have a significant complication rate, these complications are less frequent compared to PD. This may be further mitigated by the rising use of minimally invasive approaches, which have taken hold in the DP far more than pancreatic head resections [18, 19, 20]. This may increase provider discretion by making patients more likely to complete chemotherapy after surgery. Unfortunately, there remain significant disparities in systemic therapy regimens and likelihood of completing treatment by age, socioeconomic status, race, and more [21, 22]. Providers must be aware of these disparities, and possibly pursue earlier completion of chemotherapy in these patients to potentially mitigate the risk of noncompletion. In this study, when defining survival from the time of surgery, patients receiving TNT demonstrated the lowest OS, with adjustment for survival bias based on a landmark analysis, an approach commonly utilized in NCDB studies [7, 23]. This might be secondary to removal of the immortal time bias, or secondary to a signal toward the impact of completing surgery when able, yet these are purely speculation.

Finally, in a more global sense, outcomes for patients with PDAC remain quite poor overall, and the timing of chemotherapeutic regimens offered only marginal benefits in survival. Thus, additional research should focus on early surveillance of patients at high risk of PDAC including genetics, smoking history, pancreatitis, and dysplastic pancreatic cysts, with focused regimens designed to prevent rather than treat pancreatic cancer [24, 25, 26, 27, 28]. Early discovery of smaller cancers even after malignant transformation has been associated with improved outcomes [28, 29, 30]. Screening of the average risk patient is currently not recommended, but it is clear that screening techniques are improving, and we suspect that early detection and cancer prevention will provide the optimal improvement in pancreatic cancer [28, 31, 32].

This study has limitations. Most notably are the standard limitations associated with large database studies, including a lack of information regarding the reason various treatment approaches were chosen and the potential for incorrect data on a national scale. These risks are somewhat though not entirely mitigated by achieving a very large sample size in relation to this disease. Another possibly critical limitation is the potential for biases in calculated survival time introduced by various treatment sequences. For example, patients receiving TNT must have lived for a mandatory 24 weeks (12 cycles) of treatment before surgery vs. those receiving only AST, which would have the survival entirely after surgery. Conversely, beginning at surgery does not reflect the full disease course, which is the clear intention of this study, to assess the optimal treatment plan for patients with distal PDAC at the time they first present. We chose a starting point of surgical resection; however, this could also introduce some bias, equally as could starting from the time of diagnosis. We acknowledge this bias cannot be entirely overcome and for this reason, we provided both survival analyses which reach different conclusions. We prefer the approach beginning at the time of surgery because when analysis is begun at diagnosis, TNT and NAST patients benefit from both the immortal time bias and potential selection of beneficial tumor biology established by a lengthy neoadjuvant treatment period. In this regard, it is noteworthy that NAST/TNT and AST patients are somewhat heterogeneous. Patients selected for upfront surgery likely had anatomically resectable disease, whereas NAST/TNT patients were more likely to include patients who might not be for upfront resection or have locally advanced or borderline resectable tumors. These differences represent a limitation of the present study when interpreting the results. We cannot ascertain the exact chemotherapy/systemic regimens, duration, dosage, and response as this is not recorded in NCDB, which would be important information. The lack of this granular data could have played a role in the different survival observed, and the impact of this could not be established in the present study. Data in recurrence modalities and disease‐free survival are missing from this dataset which further limits interpretation. Moreover, the exact causes of death are not reported on NCBD, and this might potentially lead to bias in analyzing OS, as death may be linked to side effects of chemotherapy, surgical complications, or other factors without being directly a consequence of the disease recurrence itself. Finally, NCDB only includes Commission on Cancer Hospitals, therefore the findings of this study might not be generalizable to broader population and different healthcare systems.

## 5 Conclusion

Adjuvant systemic therapy and combined neoadjuvant and adjuvant systemic therapy were associated with marginally improved survival compared with regimens consisting of only systemic therapy preceding surgical resection for initially resectable pancreatic body/tail PDAC. Our results warrant the necessity of large multicenter studies or randomized trials to better define the role of perioperative chemotherapy regimens in distal PDAC.

## Author Contributions

**Chase J. Wehrle:** study concept, study design, data curation, investigation, collection, data analysis, interpretation, project administration, resources, validation, visualization, writing–original draft, writing–review and editing. **Jenny Chang:** writing–original draft, writing–review and editing. **Abby Gross:** writing–original draft, writing–review and editing. **Breanna Perlmutter:** writing–review and editing. **Robert Naples:** writing–review and editing. **Katherine Stackhouse:** writing–review and editing. **Toms Augustin:** writing–review and editing. **Daniel Joyce:** writing–review and editing. **Robert Simon:** writing–review and editing. **Andrea Schlegel:** writing–review and editing. **R. Matthew Walsh:** writing–review and editing. **Samer A. Naffouje:** study concept, study design, data curation, investigation, collection, data analysis, interpretation, project administration, resources, validation, visualization, writing–original draft, **Alessandro Parente:** study concept, study design, data curation, investigation, collection, data analysis, interpretation, project administration, resources, validation, visualization, writing–original draft. All authors revised and approved the manuscript.

## Conflicts of Interest

The authors declare no conflicts of interest.

## Synopsis

- We aim to provide the first large‐scale assessment of the impact of timing of peri‐operative chemotherapy on outcomes of resection of PDAC located in the pancreatic body and tail.
- Notably, the sequence of chemotherapy and surgery did not impact survival in distal PDAC.
- Providers should tailor an individualized approach designed to maximize chance of completing both treatments.

## Supporting information

Supporting information.

## Data Availability

The data are publically available through the National Cancer Database (NCDB, https://training.seer.cancer.gov/operations/standards/setters/ncdb.html).
