# Supplementary material for: Sequence of Chemotherapy May Not Impact Survival After Resection of Pancreatic Tail Adenocarcinoma
Source: J Surg Oncol. 2025 Jan 13;131(7):1362–7. doi: 10.1002/jso.28086 (PMC12186010; doi:10.1002/jso.28086)

**Supplementary table 1A**: Comparison of the treatment groups before the match.

|  | **TNT** | **NAST** | **AST** | **NAST +  AST** | **p** |
| --- | --- | --- | --- | --- | --- |
| **N** | 400 | 536 | 3,235 | 506 |  |
| **Age** | 64.6 ± 9.4 | 65.8 ± 9.9 | 66.1 ± 10.2 | 65.5 ± 9.2 | **0.027*** |
| **Sex** |  |  |  |  | 0.657 |
| Male | 185 (46.3%) | 249 (46.5%) | 1,570 (48.5%) | 249 (49.2%) |  |
| Female | 215 (53.8%) | 287 (53.5%) | 1,665 (51.5%) | 257 (50.8%) |  |
| **Race** |  |  |  |  | 0.215 |
| White | 342 (85.5%) | 455 (84.9%) | 2,695 (83.3%) | 443 (87.5%) |  |
| Black | 40 (10.0%) | 52 (9.7%) | 382 (11.8%) | 43 (8.5%) |  |
| Other | 18 (4.5%) | 29 (5.4%) | 158 (4.9%) | 20 (4.0%) |  |
| **Charlson score** |  |  |  |  | 0.098 |
| 0 | 272 (68.0%) | 324 (60.4%) | 2,042 (63.1%) | 339 (67.0%) |  |
| 1 | 89 (22.3%) | 150 (28.0%) | 848 (26.2%) | 115 (22.7%) |  |
| 2 | 20 (5.0%) | 36 (6.7%) | 235 (7.3%) | 34 (6.7%) |  |
| 3+ | 19 (4.8%) | 26 (4.9%) | 110 (3.4%) | 18 (3.6%) |  |
| **Pathologic T stage** |  |  |  |  | **<0.001*** |
| T1 | 139 (34.8%) | 145 (27.1%) | 301 (9.3%) | 123 (24.3%) |  |
| T2 | 105 (26.3%) | 147 (27.4%) | 742 (22.9%) | 147 (29.1%) |  |
| T3 | 120 (30.0%) | 213 (36.7%) | 2,030 (62.8%) | 204 (40.3%) |  |
| T4 | 20 (5.0%) | 14 (2.6%) | 69 (2.1%) | 26 (5.1%) |  |
| Tx | 16 (4.0%) | 17 (3.2%) | 93 (2.9%) | 6 (1.2%) |  |
| **Pathologic N stage** |  |  |  |  | **<0.001*** |
| N0 | 283 (70.8%) | 333 (62.1%) | 1,361 (51.1%) | 248 (49.0%) |  |
| N1 | 89 (22.3%) | 153 (28.5%) | 1,322 (40.9%) | 204 (40.3%) |  |
| N2 | 28 (7.0%) | 50 (9.3%) | 552 (17.1%) | 54 (10.7%) |  |
| **Examined nodes** | 17.7 ± 10.6 | 15.9 ± 10.6 | 15.2 ± 9.6 | 17.8 ± 9.9 | **<0.001*** |
| **Positive nodes** | 0.8 ± 1.8 | 1.0 ± 2.0 | 1.8 ± 2.6 | 1.3 ± 2.0 | **<0.001*** |
| **Margins** |  |  |  |  | **<0.001*** |
| Negative | 344 (86.0%) | 477 (89.0%) | 2,574 (79.6%) | 415 (82.0%) |  |
| Positive | 56 (14.0%) | 59 (11.0%) | 661 (20.4%) | 91 (18.0%) |  |

**Supplementary table 2**: Comparison of the treatment groups after the match.

|  | **TNT** | **NAST** | **AST** | **NAST +  AST** | **p** |
| --- | --- | --- | --- | --- | --- |
| **N** | 341 | 341 | 341 | 341 |  |
| **Age** | 64.9 ± 9.5 | 65.1 ± 9.6 | 66.0 ± 10.2 | 65.9 ± 9.1 | 0.286 |
| **Sex** |  |  |  |  | 0.995 |
| Male | 159 (46.6%) | 162 (47.5%) | 161 (47.2%) | 162 (47.5%) |  |
| Female | 182 (53.4%) | 179 (52.5%) | 180 (52.8%) | 179 (52.5%) |  |
| **Race** |  |  |  |  | 0.588 |
| White | 295 (86.5%) | 286 (83.9%) | 283 (83.0%) | 297 (87.1%) |  |
| Black | 33 (9.7%) | 35 (10.3%) | 42 (12.3%) | 29 (8.5%) |  |
| Other | 13 (3.6%) | 20 (5.9%) | 16 (4.7%) | 15 (4.4%) |  |
| **Charlson score** |  |  |  |  | 0.933 |
| 0 | 223 (65.4%) | 222 (65.1%) | 231 (67.7%) | 226 (66.3%) |  |
| 1 | 82 (24.0%) | 89 (26.1%) | 79 (23.2%) | 79 (23.2%) |  |
| 2 | 18 (5.3%) | 18 (5.3%) | 17 (5.0%) | 23 (6.7%) |  |
| 3+ | 18 (5.3%) | 12 (3.5%) | 14 (4.1%) | 13 (3.8%) |  |
| **Pathologic T stage** |  |  |  |  | 0.993 |
| T1 | 117 (34.3%) | 119 (34.9%) | 113 (33.1%) | 114 (33.4%) |  |
| T2 | 91 (26.7%) | 90 (26.4%) | 90 (26.4%) | 89 (26.1%) |  |
| T3 | 113 (33.1%) | 114 (33.4%) | 117 (34.3%) | 118 (34.6%) |  |
| T4 | 14 (4.1%) | 12 (3.5%) | 13 (3.8%) | 15 (4.4%) |  |
| Tx | 6 (1.8%) | 6 (1.8%) | 8 (2.3%) | 6 (1.8%) |  |
| **Pathologic N stage** |  |  |  |  | 0.995 |
| N0 | 228 (66.9%) | 230 (67.4%) | 223 (65.4%) | 225 (66.0%) |  |
| N1 | 87 (25.5%) | 85 (24.9%) | 92 (27.0%) | 92 (27.0%) |  |
| N2 | 26 (7.6%) | 26 (7.6%) | 26 (7.6%) | 24 (7.0%) |  |
| **Examined nodes** | 16.7 ± 10.3 | 16.7 ± 10.6 | 16.3 ± 10.6 | 16.6 ± 9.6 | 0.295 |
| **Positive nodes** | 0.9 ± 1.9 | 0.9 ± 1.8 | 0.9 ± 1.6 | 0.9 ± 1.7 | 0.997 |
| **Margins** |  |  |  |  | 0.485 |
| Negative | 291 (85.3%) | 297 (87.1%) | 287 (84.2%) | 283 (83.0%) |  |
| Positive | 50 (14.7%) | 44 (12.9%) | 54 (15.8%) | 58 (17.0%) |  |

**Supplement 3**

Kaplan-Meier analysis showed a significant reduction in median survival between groups, with the shortest median survival from time of surgery found in the TNT group (25.8 ± 2.4 months) followed y NAST (27.7 ± 1.9 months), NAST+AST (31.93 ± 2.5 months) and the longest survival from surgery in the AST group (32.53 ± 3.3 months, p=0.012). This does translate to an increase in actuarial 5-year OS from 26% in the TNT group to 33% in the AST and NAST+AST groups [**Figure**].

**Figure**: Kaplan Meier plots for overall survival in the matched groups with survival defined from the date of surgery.


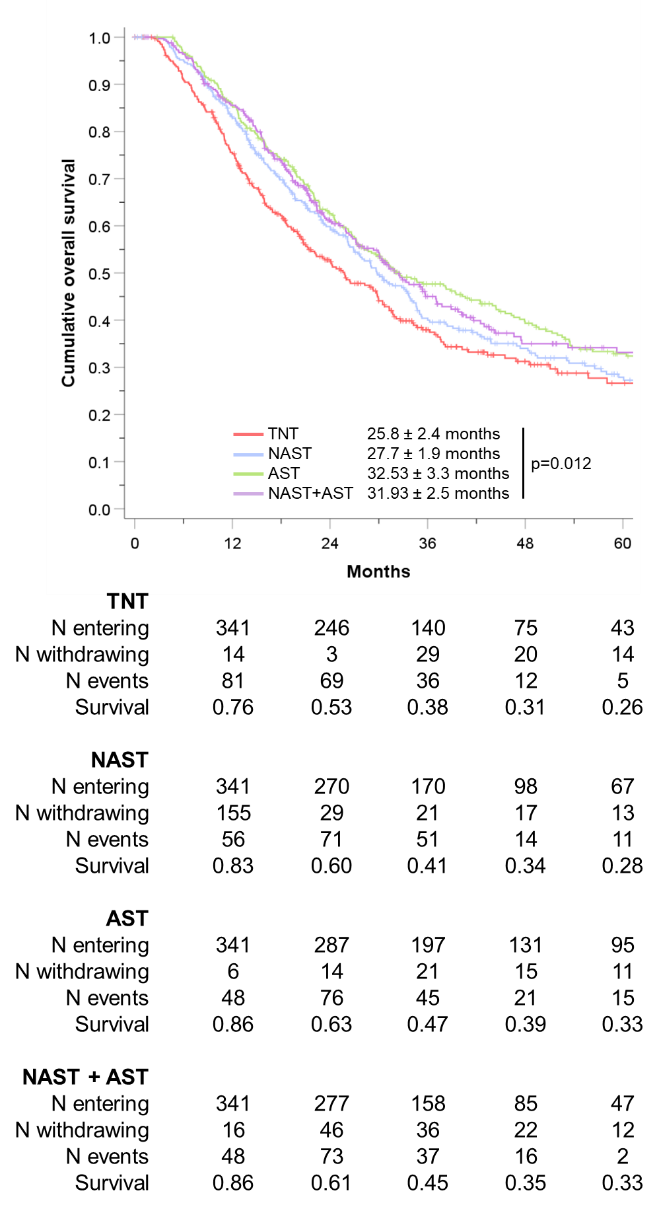

Supplement: Supplementary file 1 — Supporting information. [file JSO-131-1362-s001.docx]
